# Morphologic, phenotypic, and genotypic similarities between primary tumors and corresponding 3D cell cultures grown in a repeatable system—preliminary results

**DOI:** 10.1186/s12917-023-03834-7

**Published:** 2023-12-09

**Authors:** Luisa Vera Muscatello, Stella Frabetti, Giancarlo Avallone, Francesca Gobbo, Arianna Pasquini, Giulia D’Annunzio, Luciano Pisoni, Laura Marconato, Rossella Terragni, Dario De Biase, Olivia Candini, Giuseppe Sarli

**Affiliations:** 1 Department of Veterinary Medical Sciences, University of Bologna, I-40064 Ozzano dell’Emilia, BO Italy; 2 Evotec (Modena) S.r.l., I-41036 Medolla, MO Italy; 3 Experimental Zooprophylactic Institute of Lombardia and Emilia Romagna, I-25124 Brescia, Italy; 4 Pet Care, I-40133 Bologna, Italy; 5 Department of Pharmacy and BiotechnologyUniversity of Bologna, I-40127 Bologna, Italy

**Keywords:** 3D cell culture, Dog, Spontaneous Neoplasms, Tumor cells morphology, Immunophenotypes, Genetic profile, Repeatable system

## Abstract

**Background:**

Three-dimensional (3D) cell cultures are the new frontier for reproducing the tumor micro-environment in vitro. The aims of the study were (1) to establish primary 3D cell cultures from canine spontaneous neoplasms and (2) to demonstrate the morphological, phenotypic and genotypic similarities between the primary canine neoplasms and the corresponding 3D cultures, through the expression of tumor differentiation markers.

**Results:**

Seven primary tumors were collected, including 4 carcinomas and 3 soft tissue sarcomas. 3D cell cultures reproduced the morphological features of the primary tumors and showed an overlapping immunophenotype of the primary epithelial tumors. Immunohistochemistry demonstrated the growth of stromal cells and macrophages admixed with the neoplastic epithelial component, reproducing the tumor microenvironment. Mesenchymal 3D cultures reproduced the immunophenotype of the primary tumor completely in 2 out of 3 examined cases while a discordant expression was documented for a single marker in one case. No single nucleotide variants or small indel were detected in *TP53* or *MDM2* genes, both in primary tumors and in 3D cell cultures specimens. In one sample, *MDM2* amplicons were preferentially increased in number compared to *TP53* ones, indicating amplification of *MDM2*, detectable both in the primary tumor and in the corresponding cell culture specimen.

**Conclusion:**

Here we demonstrate a good cell morphology, phenotype and genetic profile overlap between primary tumors and the corresponding 3D cultures grown in a repeatable system.

**Supplementary Information:**

The online version contains supplementary material available at 10.1186/s12917-023-03834-7.

## Background

Cell-based assays are key tools to assess the potential efficacy of new compounds in drug discovery assessment. Increasing evidence indicates that 3D cell culture systems reproduce more accurately tissue microenvironment compared to 2D cultures. The spatiality of 3D cultures is crucial for spatial organization of surface receptors involved in cell-to-cell interactions (e.g., neoplastic to stromal cells), providing also physical constraints to neoplastic cells, thus more closely mimicking in vivo conditions [1]. The spatial and physical features of 3D cultures affect signal transduction, and ultimately influence gene expression and cell behavior inasmuch as cell responses in 3D cultures better mimic those observed in vivo compared to 2D cultures [2–4].

Furthermore, 3D culture models allow cells to develop a morphology similar to the natural in vivo counterpart [5, 6] and a cell population heterogeneity including proliferating, quiescent, apoptotic, hypoxic and necrotic cells [5, 7].

Such diversity recapitulates more closely the in vivo tumor microenvironment. All the above explains the observation that cellular processes in 3D cultures closely emulate those in vivo [1]. This evidence indicates that the 3D environment is critical for the preservation of the transcriptional and translational cell activities and thus, the gene and protein expression profiles, providing an environment where neoplastic cells more likely behave [7] as they would in vivo. While many studies on 3D cultures have been performed with established cancer cell lines, more recently, the use of patient-derived primary tumor cells has been reported [1, 8]. Although this technology has not been optimized, these studies have shown the potential of 3D cultures of patient-derived primary tumor cells for revealing cancer biology and developing new therapeutic approaches [9].

One limit of this approach is the plethora and complexity of the available systems for 3D growths [1] and the lack of comparable protocols among studies, which hinder standardization and hamper the acquisition of reliable, reproducible, and unequivocally interpretable results. Today, the availability of ready-to-use devices for 3D culture generation offers the opportunity to improve reproducibility and reliability, thereby opening the door to a more approachable and widespread usage of 3D cultures.

The application of such methods to canine neoplasms has two main facets: (1) many canine tumors are spontaneous models for the human counterparts having translational relevance; (2) cancer treatment in veterinary medicine is an essential part in comprehensive pet primary care with therapeutic approaches based on tumor type, histological grade and clinical stage, including surgery, radiation therapy, chemotherapy and immunotherapy [10]. Therefore, the study of tumors through cell cultures and the use of new molecules in vitro lay the foundations for a personalized therapeutic approach in the management of animal tumors.

Canine cancer cell have been cultured most commonly as 2D cultures obtained from several types of neoplasms, including soft and hard tissue sarcoma [11–14] and different histotypes of carcinoma [15–17] to demonstrate feasibility, obtain abundant neoplastic cell material for genetic studies and for therapy efficacy assessment. For osteosarcoma, lymphoma [18, 19] and mammary carcinoma, [17] 3D culture systems have been recently used to study specific target therapies and demonstrate the importance of the tumor specific stromal microenvironment. These studies have demonstrated the better performances and the essential role of 3D culture systems for the understanding of treatment response and the emergence of chemo-resistant clones. More so, 3D systems allowing interactions between the tumor microenvironment and the corresponding neoplastic cells have proven essential in designing experimental approaches to personalized medicine and predicting the effect of different drugs also in dogs [11, 18, 20].

The study of specific canine tumors is considered useful as they represent spontaneous models for the human counterparts. Compared to xenograft or genetically modified murine models, the dog shares with humans more than ~ 650 Mb of ancestral sequences; furthermore, canine DNA and protein sequences are more similar to humans compared to rodents [21]. According to genetic studies, the dog is a good candidate species because of a well-established literature with consistent data, providing a model with higher probability to bear genetic anomalies in the primary tumor that may be demonstrated specifically in the corresponding 3D culture.

The aims of the current study were: (1) to collect and establish primary 3D cell cultures from canine spontaneous neoplasms, by a small and fiber-based bioreactor developed to recreate a 3D tissue-like structure in a closed system, which integrates a 3D scaffold able to sustain growth of normal and neoplastic cells within a biologically relevant environment; (2) to demonstrate the morphologic, phenotypic and genetic similarities between the primary canine epithelial and mesenchymal neoplasms and the corresponding 3D cultures, through the expression of the same tumor markers.

## Results

### Caseload and histologic diagnosis

Seven primary tumors were collected, including 4 carcinomas and 3 soft tissue sarcomas.

Among carcinomas, there were 2 grade 2 pulmonary carcinomas (papillary carcinoma and squamous cell carcinoma with papillary and lepidic differentiation, respectively), one of which having nodal metastatic disease; 1 follicular-compact thyroid carcinoma with nodal metastasis; and 1 sebaceous carcinoma.

Among sarcomas, there were 3 grade 2 perivascular wall tumors. There were no lymph node metastases.

### Morphologic comparison between primary tumors and 3D cell cultures

A quality control was carried out by using a cellularity scoring system. Cellularity was generally optimal with moderate (3 out of 7 cases), high (1 out of 7 cases) and very high (1 out of 7 cases) scoring, two cases had good (1 out of 7 cases) to scant (1 out of 7 cases) cellularity (Table 1). In cultures with moderate to high scoring, cellularity was continuous along the scaffold, with formation of multilayers.

**Fig. 1:**
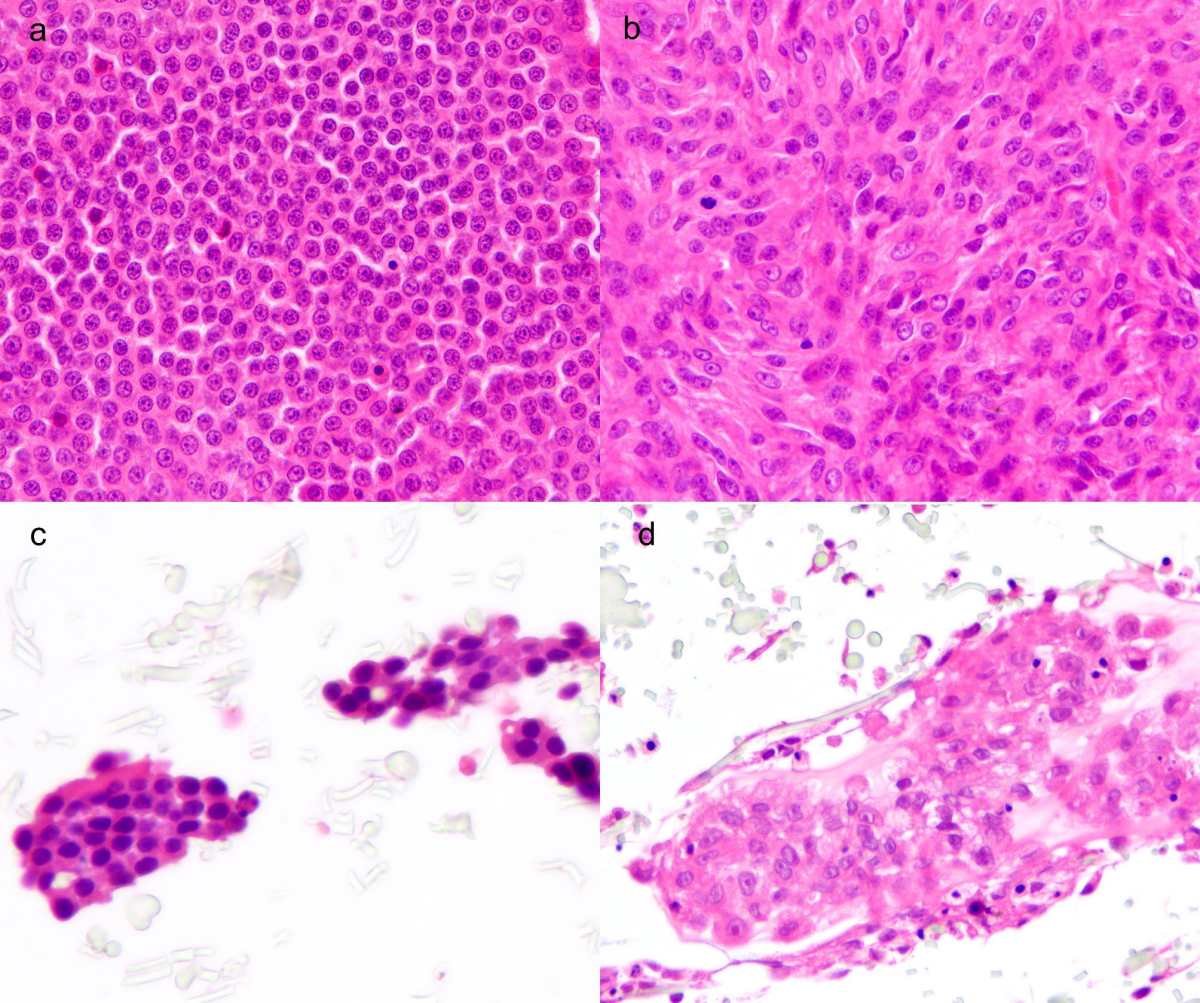
Morphology of primary tumors and the respective 3D cultures, hematoxylin and eosin. (**a**) Follicular-compact thyroidal carcinoma: solid lobules of polygonal cells. (**b**) Perivascular wall tumor, storiform pattern of spindle cells. (**c**) 3D culture of follicular-compact carcinoma: moderate cellularity (score 3) forming solid aggregates of polygonal cells. (**d**) 3D culture of perivascular wall tumor: very high cellularity (score 5) forming solid whorls of spindle cells

Cultures reproduced the morphologic patterns found in the primary tumors, forming papillae in pulmonary carcinomas, solid lobules in thyroid and sebaceous carcinomas, bundles and clusters in perivascular wall tumor. Examples of epithelial and mesenchymal histological patterns, both in the primary tumor and 3D cell culture, are shown in Fig. 1.

Cell shape was also preserved, maintaining a polygonal cellular and nuclear shape in carcinomas and spindle to polygonal shape in perivascular wall tumors. The degree of differentiation in cultured cells was enough to mimic, in addition to the shape and nuclear-cytoplasmic ratio, also other morphologic features that suggest a specific histotype, such as cytoplasmic vacuolization in the case of sebaceous carcinoma. Residues of matrix fiber are visible as light yellow stretched (Fig. 1c and d).

### Immunophenotypic comparison between primary tumors and 3D cell cultures

**Fig. 2:**
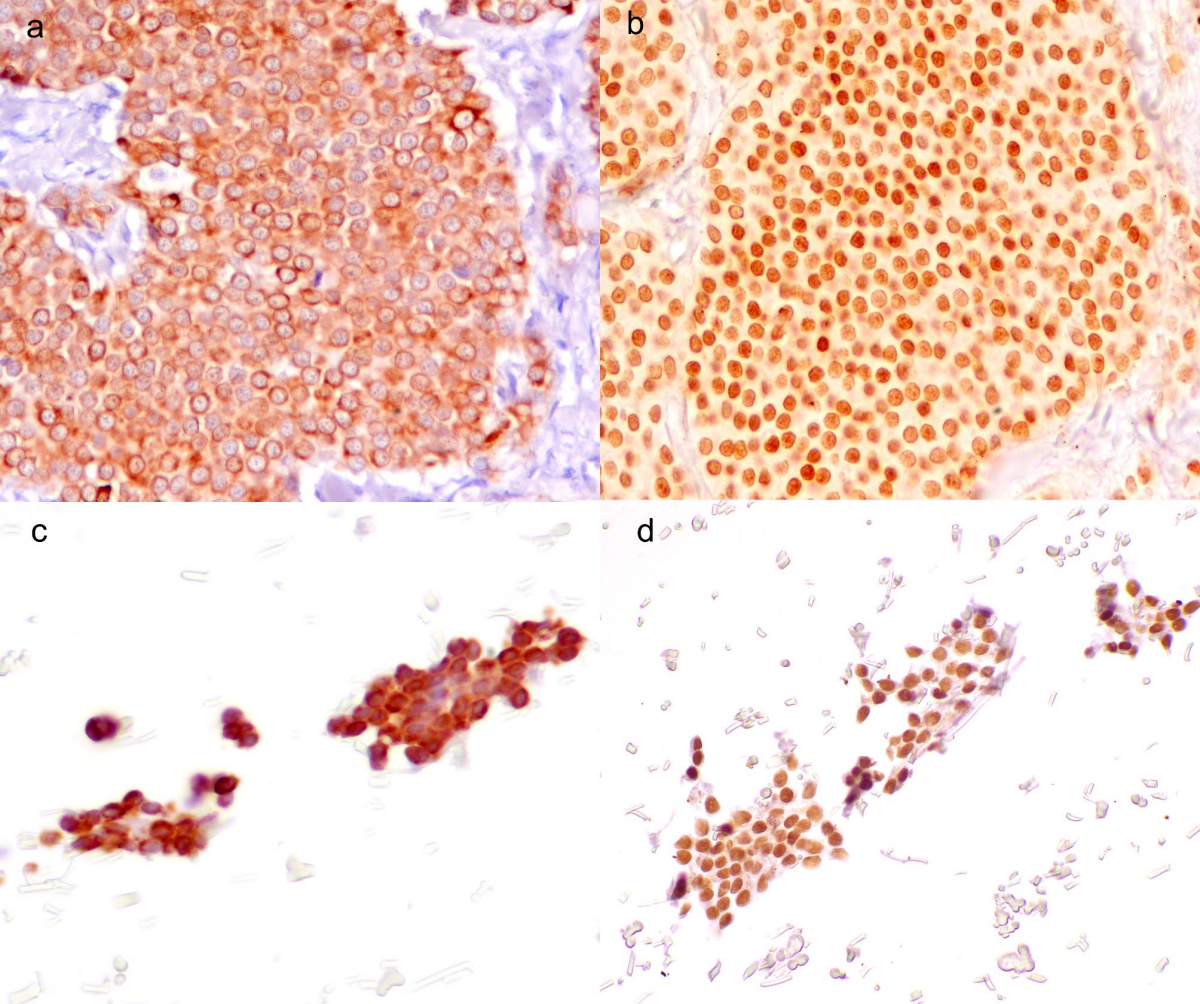
Immunophenotype of primary epithelial tumor (follicular-compact thyroidal carcinoma) and the respective 3D cultures, diaminobenzidine. (**a**) Follicular-compact thyroidal carcinoma, pancytokeratin: solid lobules of neoplastic cells with moderate diffuse cytoplasmic immunoexpression. (**b**) Follicular-compact thyroidal carcinoma, TTF1: solid lobules of neoplastic cells with intense diffuse nuclear immunoexpression. (**c**) 3D culture of follicular-compact carcinoma, pancytokeratin: solid aggregates of neoplastic cells with moderate diffuse cytoplasmic immunoexpression. (**d**) 3D culture of follicular-compact carcinoma, TTF1: solid aggregates of neoplastic cells with intense diffuse nuclear immunoexpression

Both pulmonary carcinomas and the one thyroid carcinoma consistently expressed pancytokeratin and TTF1 with intense diffuse cytoplasmic and nuclear immunoexpression, respectively (Fig. 2).

The sebaceous carcinoma diffusely expressed pancytokeratin at the cytoplasmic level. Numerous vacuolized cells, ascribable to macrophages, negative for pancytokeratin and positive for CD18, were observed in the stroma. In all carcinomas, the stromal cells markedly expressed vimentin, with diffuse and intense cytoplasmic staining.

Among carcinomas, the 3D cultures faithfully reproduced the immunophenotype of the primary tumor in all cases. Notably, supporting stromal cells and macrophages were also present in 3D cultures, reproducing the tumor microenvironment (Supplementary Fig. 1).

**Fig. 3:**
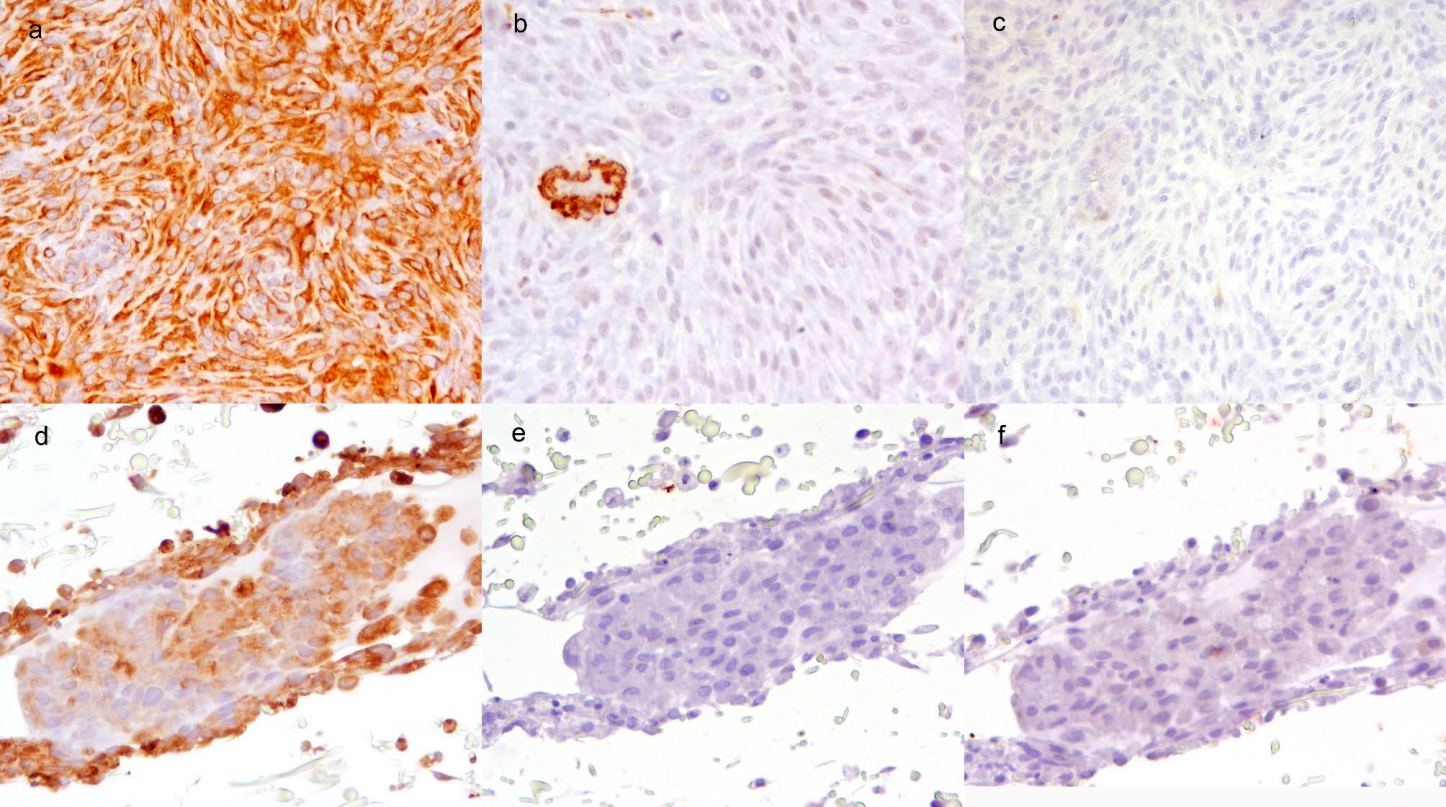
Immunophenotype of primary mesenchymal tumor (perivascular wall tumor) and the respective 3D cultures, diaminobenzidine. (**a**) Perivascular wall tumor, vimentin: solid whorls of neoplastic cells with intense diffuse cytoplasmic immunoexpression. (**b**) Perivascular wall tumor, actin: negative expression by neoplastic cells and internal positive control in the vessel wall. (**c**) Perivascular wall tumor, calponin: negative expression by neoplastic cells. (**d**) 3D culture of perivascular wall tumors, vimentin: solid whorls of neoplastic cells with intense diffuse cytoplasmic immunoexpression in the periphery of the aggregate and less intense labeling in the internal portion penetrant in the scaffold. (**e**) 3D culture of perivascular wall tumor, actin: negative expression by neoplastic cells. (**f**) 3D culture of perivascular wall tumor, calponin: negative expression by neoplastic cells

All perivascular wall tumors showed moderate and diffuse cytoplasmic vimentin expression; among them, one case also showed cytoplasmic actin expression in almost 50% of the cells. Calponin was negative in all cases. The 3D cultures of the corresponding mesenchymal tumors completely reproduced the phenotype of the primary tumor in two out of three examined cases (Fig. 3). In the third case (ID6), although there was a concordant expression of vimentin and calponin between primary tumor and 3D culture, discordant expression was observed for actin, since it was present in the primary tumor and not in the cell culture.

The immunohistochemical results and cellularity scoring are summarized in Table 1.

**Table 1:** Immunomorphological comparison between primary tumors and 3D cell cultures

ID	Anatomic site	Histological diagnosis	3D cell culture cellularity scoring (1–5)		Primary tumor immunophenotype	3D cell culture immunophenotype
1	Lung	Papillary carcinoma		2	Panck + TTF1+	Panck + TTF1+
2	Lung	Squamous carcinoma with papillary and lepidic differentiation		2	Panck + TTF1+	Panck + TTF1+
3	Skin	Epitheliomatous sebaceous carcinoma		4	Panck+	Panck+
4	Thyroid gland	Follicular-compact carcinoma		4	Panck + TTF1+	Panck + TTF1+
5	Skin	Perivascular wall tumor		3	Vim + act- calp-	Vim + act- calp-
6	Skin	Perivascular wall tumor		3	Vim + act + calp-	Vim + act- calp-
7	Skin	Perivascular wall tumor		5	Vim + act- calp-	Vim + act- calp-

vim = vimentin; act = actin; calp = calponin; panck = pancytokeratin

### Sequence analysis in primary tumors and 3D cell cultures using next-generation sequencing

**Fig. 4:**
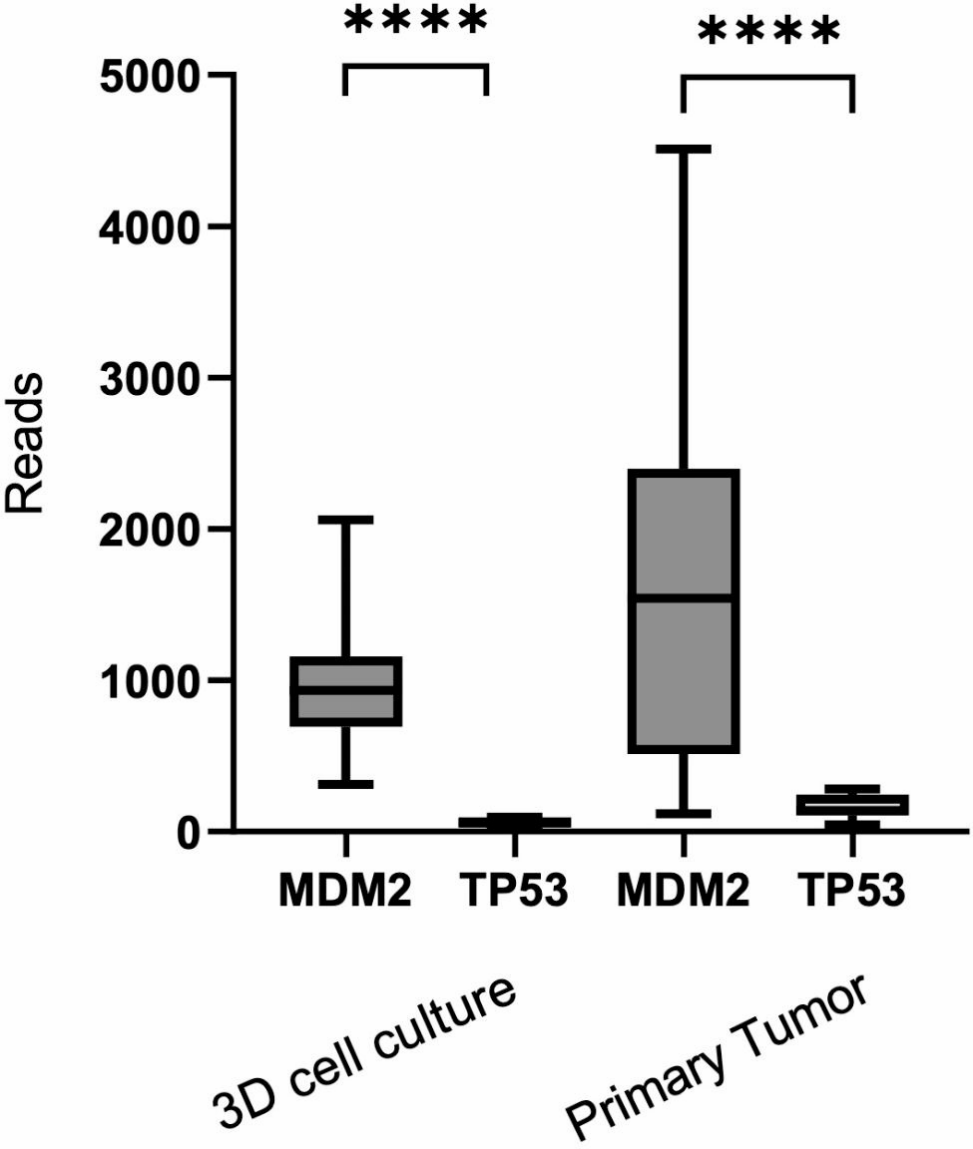
Amplicon coverage in *MDM2* and *TP53* in 3D cell culture and in primary tumor specimens (Case 1, Table 2). *P*-value: Mann-Whitney test

No single nucleotide variants (SNV) or small indel were detected in *TP53* or *MDM2* genes, both in primary tumors and in 3D cell culture specimens (Tables 2 and 3). The mean coverage in cell culture specimens ranged from 144 to 613 reads and from 30 to 1190 for primary sarcoma samples (Table 2), and from 72 to 483 reads in cell culture specimens and from 1105 to 1763 for primary carcinoma samples (Table 3). One case was not evaluable both in primary and in 3D cell culture specimens: this data highlights that ex vivo and 3D-cell are equivalent to each other. Intriguingly in one sample, both in primary tumor and cell culture specimens, *MDM2* amplicons were preferentially amplified if compared to *TP53* ones (ratio “mean amplicon coverage *MDM2*/ mean amplicon coverage *TP53*” = 16.5 and 10.7, respectively) (Table 2; Fig. 4). This data may indicate amplification of *MDM2*, detectable both in primary tumor and in cell culture specimens. No putative *MDM2* amplification was detected in carcinoma samples (Table 3).

**Table 2:** NGS data of primary tumors and 3D cell cultures. In bold: ratio *MDM2*/*TP53* suggestive of *MDM2* amplification

Case	Specimen	*TP53*	*MDM2*	Mean coverage (reads per amplicon)	Mean coverage *MDM2*	Mean coverage *TP53*	Ratio (*MDM2*/*TP53*)
1	*Primary Tumor*	WT	WT	1112	1685	157	**10.7**
	3D cell culture	WT	WT	613	990	60	**16.5**
2	*Primary Tumor*	WT	WT	1105	1185	1000	1.2
	3D cell culture	WT	WT	144	151	132	1.1
3	*Primary Tumor*	WT	WT	1763	1947	1565	1.2
	3D cell culture	WT	WT	711	806	590	1.4

WT = Wild Type

**Table 3:** NGS data of primary tumors and 3D cell cultures from carcinoma samples

Case	Specimen	*TP53*	*MDM2*	Mean coverage (reads per amplicon)	Mean coverage *MDM2*	Mean coverage *TP53*	Ratio (*MDM2*/*TP53*)
1	*Primary Tumor*	WT	WT	1190	1446	863	1.37
	3D cell culture	WT	WT	483	494	468	1.03
2	*Primary Tumor*	WT	WT	30	15	49	0.61
	3D cell culture	WT	WT	72	72	72	1.0
3	*Primary Tumor*	WT	WT	591	595	585	1.0
	3D cell culture	WT	WT	340	367	306	1.1
4	*Primary Tumor*	NE	NE	/	/	/	/
	3D cell culture	NE	NE	/	/	/	/

WT: Wild Type; NE: Not Evaluable

## Discussion

3D cell cultures are techniques that accurately simulate cell growth, including neoplastic cells, offering significant opportunities for cancer treatment research. They enable the investigation of molecular therapeutic targets and their pharmacological inhibition. Indeed, 3D cultures represent valuable simulators of cancer tissues, as they exhibit similar growth and treatment response patterns [1].

However, there are challenges to be addressed, as the reproducibility of 3D cultures depends on many variables, including the substrate in which cells grow, the proper phase of growth and the variability of cancer features.

The model applied in this study reduces the substrate-related variables, since a small bioreactor, integrating a 3D synthetic, inert, and biocompatible scaffold, was used to favor reproducible cell colonization and growth. Due to the high amount of cells, real-time monitoring of cells viability and cell number estimation with Real-Time Glo (Promega) (as reported in Candini et al. 2019) [23] is not possible. However, the scope of this work does not require this specific type of evaluation. Histology, immunohistochemistry and NGS provide evidence regarding cell density, viability, immunophenotype and genetic derangement, aiming primarily to compare the 3D culture with its original tumor counterpart.

The growth phase can be controlled by stimulating, with appropriate growth factors, only the component of interest (epithelial if the growth of carcinoma is to be stimulated, mesenchymal if of sarcoma), generating an in vitro model where tumor progression acceleration may reveal the mechanisms underlying neoplastic transformation for a drug discovery approach.

In the present study, the fiber-based bioreactor facilitated the 3D growth of tumor cells originating from both epithelial and mesenchymal tissues in dogs. This aligns with findings in humans, where 3D growth has been observed in pancreatic, breast and lung adenocarcinoma, as well as in Ewing’s sarcoma [23].

Furthermore, the morphologic and immunophenotypic analogies between 3D growth and primary tumor, combined with the rapid establishment of primary cultures, reduced the quantity of culture medium and growth factors, being time- and cost-effective. Compared to 2D cultures and even to animal models, 3D cultures provide a more reliable tool in predicting how drug treatments will affect patients [22].

Animal models might not consistently bridge the differences among species under study. Furthermore, 3D cells culture serves as a straightforward, rapid, and cost-effective tool, reducing the need for extensive and expensive animal testing. One major limitation of 2D cell cultures is their inability to replicate the three-dimensional microenvironment of biological tissues found in vivo. In contrast, 3D cell cultures can replicate the extracellular matrix, offering a more realistic mimicry of natural tissues. This resemblance extends beyond cell-to-cell interactions to include cell-extracellular matrix interactions [22].

Most of the in vitro knowledge regarding spontaneous tumors in small animals comes from the study of 2D cell cultures or of the tumor itself [1]. However, it is well known that the monolayer 2D growth is represented by neoplastic cells having unlimited access to the ingredients of the medium, including oxygen, nutrients and signal molecules, which does not mimic a natural neoplastic mass. An additional important drawback is due to the fact that cancer development and progression rely not only on the main cell population, but also on the interactions with the associated stroma, intended as the matrix and stromal cells. When neoplastic cells are placed back into an in vivo environment (i.e., an animal model) or are grown in a 3D system, proliferation and gene/protein expression are much closer to the ones occurring within the tumor than those grown in a 2D systems [1].

In our study, it has been shown that morphology and immunophenotype of 3D cell cultures are able to adequately replicate the growth of canine primary carcinomas and soft tissue sarcomas, not only by the expression of the primary tumor markers, but also by re-creating a tumor microenvironment, characterized by stromal fibroblasts in all the epithelial tumor cultures and inflammatory cells (macrophages in one case).

While the expression of differentiation markers between primary tumors and 3D growths was overall concordant, in one sarcoma case actin was expressed by the primary tumor only. This discrepancy may be due to a de-differentiation in cell cultures rather than to the selective growth of cells negative for that marker, which represented half of the population in the primary tumor.

At the genetic level, no sequence alterations were found nether in 3D cell culture nor in primary tumor samples. Intriguingly, comparing the *MDM2* amplicon coverage value with that of *TP53*, in one case we observed a preferential amplification in *MDM2* amplicons, hypothesizing a possible *MDM2* amplification, both in 3D cell culture and in primary tumor specimens from the same sample. Indeed, in the other cases, the amplicons ratio (*MDM2* coverage:*TP53* coverage) was near 1, and also in these samples, the ratio was similar both in 3D cell culture and in primary tumor specimens.

As well as morphologic and phenotypic similarities, also genomic data are concordant between primary tumours and the corresponding 3D cell cultures, which can be therefore considered a suitable substrate for biobanking even after formalin-fixation and paraffin embedding.

In oncology, several diagnostic tools are available to evaluate the expression of molecules aimed at tailoring treatment, thereby providing a precision approach. However, many of these tests are based on the evaluation of fixed tissues where cells viability is lost, leading to the impossibility to investigate cell function during tumor growth and progression. Being the cell-to-cell and cell-to-extracellular matrix interaction a fundamental condition for more representative tumor studies, we investigated and demonstrated the reproducibility of morphologic, immunophenotypic and tumor microenvironment aspects in vitro models using 3D cultures obtained from canine carcinomas and soft tissue sarcomas. Furthermore, in this in vitro model, a genetic similarity was demonstrated with parallel amplicon coverage in primary tumor and corresponding cell culture.

## Conclusions

The results of this investigation, although preliminary, highlight the advantage to obtain in a short time a 3D model that closely mimick the primary tumor counterpart offering a methodology for precision medicine purposes in both veterinary and human tumor pathology, possibly providing a relevant alternative to animal models.

## Materials and methods

### Caseload

With prior owners’ informed consent, tumor samples were collected from dogs undergoing surgery at the University Veterinary Hospital of the Department of Veterinary Medical Sciences University of Bologna and the Veterinary Clinic “Pet Care” in Bologna. The study did not fall within the application areas of Italian Legislative Decree 26/2014 which governs the protection of animals used for scientific or educational purposes; therefore, ethical approval was waived for this study. Dogs were treated according to the current standards.

Pre-operative cytologic diagnosis was obtained to select the tumor of interest. The selection criterion was an epithelial or mesenchymal origin of the tumors. Surgical specimens were sampled for both the 3D cell culture production (1 g of tumoral tissue) and routine histologic examination (all remaining tumoral tissue), therefore small tumors with insufficient amount of tissue for both analyzes were excluded.

### 3D culture production

The tumor tissue specimens were transferred from the veterinary clinic/hospital to the laboratory of processing using the MACS Storage solution (Miltenyi Biotec) to better preserve the tissue viability. The tumor samples were first washed with Phosphate Buffered Solution (Gibco) and cut in small pieces using surgical scalpel and tweezers. Then, the tumor specimens were processed to obtain single cells using the Tumor Dissociation Kit (Miltenyi Biotec) with GentleMACS Octo Dissociators instrument with heaters (Miltenyi Biotec). The single cells obtained from the dissociation were filtered with MACS SmartStrainer 70 μm (Miltenyi Biotec) and washed with 20 mL of DMEM or RPMI medium as indicated in the Tumor Dissociation Kit datasheet. Then, the cell suspension was centrifuged at 300 g for 7 min, the supernatant was removed and the cell pellet was resuspended in an appropriate volume of culture medium for cell count. Trypan Blue staining (Gibco) was used for cell count and to check cell viability. The 3D bioreactor [23] (VITVO®, Rigenerand srl, Modena) was first primed with cell culture medium alone to ensure the complete wetting of 3D matrix. Then, 10–15 × 10^6^ primary tumor cells were suspended in 1.4 mL of culture media and injected into the system with a luer-lock syringe. Cells were cultivated for 7–10 days at 37 °C, 5% CO_2_ with media change every 48 h and using specific culture medium and growth factors to favor a fast and standardized selection of sarcomatous or carcinomatous tumor cells.

Cell number, the days of culture and the culture media were defined during preliminary experiment; RPMI medium (Gibco) supplemented with 10% Fetal Bovine Serum (Gibco), 1% Penicillin-streptomycin (Gibco), 1% Glutamine (Gibco), EGF and FGF-7 (KGF) (STEMCELL technologies) 10 ng/mL was selected for carcinoma, DMEM medium (Gibco) supplemented with 10% Fetal Bovine Serum (Gibco), 1% Penicillin-streptomycin (Gibco), 1% Glutamine (Gibco), b-FGF and TGF-β 10 ng/mL was selected for sarcoma cultivation.

From each tumor sample the generated 3D culture was then formalin fixed, directly injecting formalin inside the device, and, after an incubation of 24 h at room temperature, further processed to obtain the paraffin block.

### Histology and 3D culture embedding

Fresh tumor samples were formalin fixed-paraffin embedded and routinely stained with hematoxylin and eosin. Tumor grades were applied according to the histotype [24, 25].

**Fig. 5:**
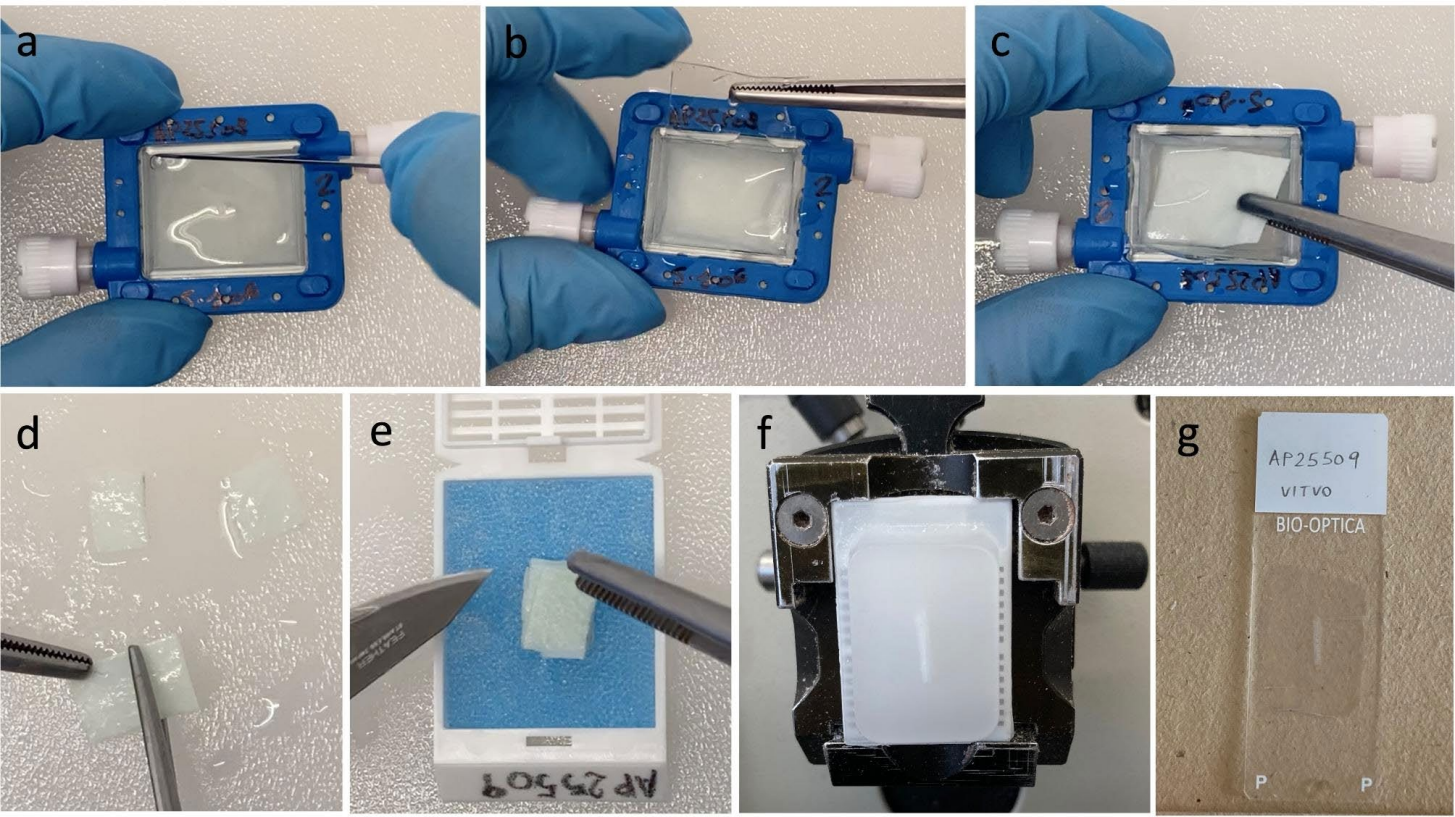
3D culture embedding for histology. Keeping the loading side up, the chamber was open with a blade (**a** and **b**) and the matrix was extracted (**c**). The matrix was sectioned in 4 fractions (**d**). The 4 fractions were allocated one above the other in the histology cassette creating a sandwich (**e**). The culture matrix was included with vertical orientation (**f**), by this way a longitudinal section of the 4 matrix sheets were allocated on a glass slide (**g**)

Regarding the 3D culture, the cell culture matrix was recovered from the VITVO device cutting the transparent oxygenation membrane with a surgical scalpel (Fig. 5a, b, c) keeping the cell loading side facing up. Then the matrix was first cut into two halves and these two portions were closed like a book facing the loading side; at this point it was cut again into other two halves (Fig. 5d), obtaining a sandwich that was allocated in a biocassette for histology (Fig. 5e). The matrix was embedded in bio-agar gel (Bio-Optica 05-9803 S) for 30 min until solidification was achieved; then it was routinely processed overnight and included in paraffin blocks (Fig. 5f), cut into 3-micron sections (Fig. 5g) and stained with hematoxylin and eosin.

Culture cellularity was assessed with a 5-tier scoring system evaluated in the most cellular area, at high power field (HPF) with an area of 0.237 mm^2^:

- 1 (scant): < 20 cells per HPF
- 2 (good): 20–39 cells per HPF
- 3 (moderate): 40–59 cells per HPF
- 4 (high): 60–79 cells per HPF
- 5 (very high): > 80 cells per HPF

### Immunohistochemistry

Serial 3-micron-thick sections of primary tumors and cell cultures were cut, and immunohistochemistry was performed simultaneously. Sections were dewaxed and rehydrated. Endogenous peroxidase was blocked by immersion in 3% H_2_O_2_ in methanol for 30 min at room temperature. Heat induced antigen retrieval was performed for 10 min in microwave oven at 750 W, followed by cooling at room temperature for 20 min. Tissue sections were incubated, for 2 h at 37 °C, with primary antibodies (against -pancytokeratin, -TTF1, -CD18 and vimentin for carcinoma and against -actin, -vimentin and -calponin for sarcomas). Binding sites were revealed by secondary biotinylated antibody (dilution 1:200) and amplified using a commercial avidin-biotin-peroxidase kit (VECTASTAIN; ABC Kits, Peterborough, UK). The chromogen 3,3-diaminobenzidine 0.05% was used and slides were counterstained with Mayer’s hematoxylin. Antibodies details are summarized in Supplementary Table 1.

### Next-generation sequencing

*TP53* and *MDM2* genes were investigated based on their role in the tumorigenesis in human and dog [26, 27]; the protocol for this analysis has been validated for another ongoing study on canine sarcomas (unpublished data).

DNA was extracted from two 10-µm thick tissue sections, both starting from primary tumors and 3D cell culture specimens. The representative area was scratched manually, and DNA extraction was performed using the Quick Extract FFPE Kit (Lucigen, LGC Biosearch Technologies, Hoddesdon, UK). DNA was quantified using the Qubit fluorometer (Thermo Fisher Scientific, USA).

DNA was amplified using a laboratory-developed (Molecular Pathology Laboratory of the Policlinico Sant’Orsola-Malpighi) NGS panel. The panel was designed using the Ion AmpliSeq Designer Tool (Thermo Fisher Scientific) and allows to amplify and sequence all the CDS (Coding Sequence) of the *TP53* gene (start chr5:32560598; end chr5:32565670) and *MDM2* gene (start chr10: 10,936,612; end chr10: 10,962,528) (reference: *CanisFamiliaris 3*). The panel included a total of 48 amplicons (panel size: 4.73 kb), with a length between 125 and 175 bp. The sequencing was performed using the GeneStudio S5 Sequencer (Ion 530™ Chip) and the results were analyzed using the Golden Helix Genome Browse tool (https://www.goldenhelix.com/products/GenomeBrowse/index.html).

### Electronic supplementary material

Below is the link to the electronic supplementary material.

Supplementary Material 1

Supplementary Material 2

## Data Availability

Sequencing data generated and analysed during the current study are available in the NCBI—Sequence Read Archive (SRA) (PRJNA947486) and at the following: https://www.ncbi.nlm.nih.gov/sra/PRJNA947486.

## List of abbreviations

3D: Three-dimensional
TP53: Tumor protein p53
MDM2: Murine double minute-2
TTF1: Transcriptional thyroid factor 1
CD18: Cluster of differentiation 18
PANCK: Pancytokeratin
ACT: Actin
CALP: Calponin
SNV: Single Nucleotide Variants
WT: Wilde Type
