# Supplementary material for: Morphologic, phenotypic, and genotypic similarities between primary tumors and corresponding 3D cell cultures grown in a repeatable system—preliminary results
Source: BMC Vet Res. 2023 Dec 9;19:263. doi: 10.1186/s12917-023-03834-7 (PMC10709889; doi:10.1186/s12917-023-03834-7)
Supplement: Supplementary file 1 — Supplementary Material 1 [file 12917_2023_3834_MOESM1_ESM.docx]

**Supplementary Table 1.** Immunohistochemical specification and antibody details.

| **Antibody** | **Clone** | **Manufacturer** | **Dilution** | **Antigen Retrival** |
| --- | --- | --- | --- | --- |
| Pancytokeratin | AE1/AE3 | DAKO Glostrup, Denmark | 1: 300 | Citrate buffer pH 6.00 in microwave at 750 W for 10 min |
| TTF1 | 8G7G3/1 | DAKO Glostrup, Denmark | 1:1000 | EDTA buffer pH 8.00 in microwave at 750W for 20 min |
| CD18 | CA16.3C610(ff) | Leucocyte’s antigen laboratory, UC Davis | 1:20 | Trypsin 0.05% pH 7.6 at 37°C for 15 min |
| Vimentin | V9 | DAKO Glostrup, Denmark | 1:600 | Citrate buffer pH 6.00 in microwave at 750 W for 10 min |
| Actin | 1A4 | Santa Cruz Biotechnology, Dallas, USA | 1:450 | Citrate buffer pH 6.00 in microwave at 750 W for 10 min |
| Calponin | CALP | DAKO Glostrup, Denmark | 1:2000 | Proteinase K 0.04 mg/ml pH 7.6 at 37°C for 10 min and citrate buffer pH 6.00 in microwave at 750 W for 10 min |
