# Supplementary material for: Morphologic, phenotypic, and genotypic similarities between primary tumors and corresponding 3D cell cultures grown in a repeatable system—preliminary results
Source: BMC Vet Res. 2023 Dec 9;19:263. doi: 10.1186/s12917-023-03834-7 (PMC10709889; doi:10.1186/s12917-023-03834-7)
Supplement: Supplementary file 2 — Supplementary Material 2 [file 12917_2023_3834_MOESM2_ESM.docx]

**
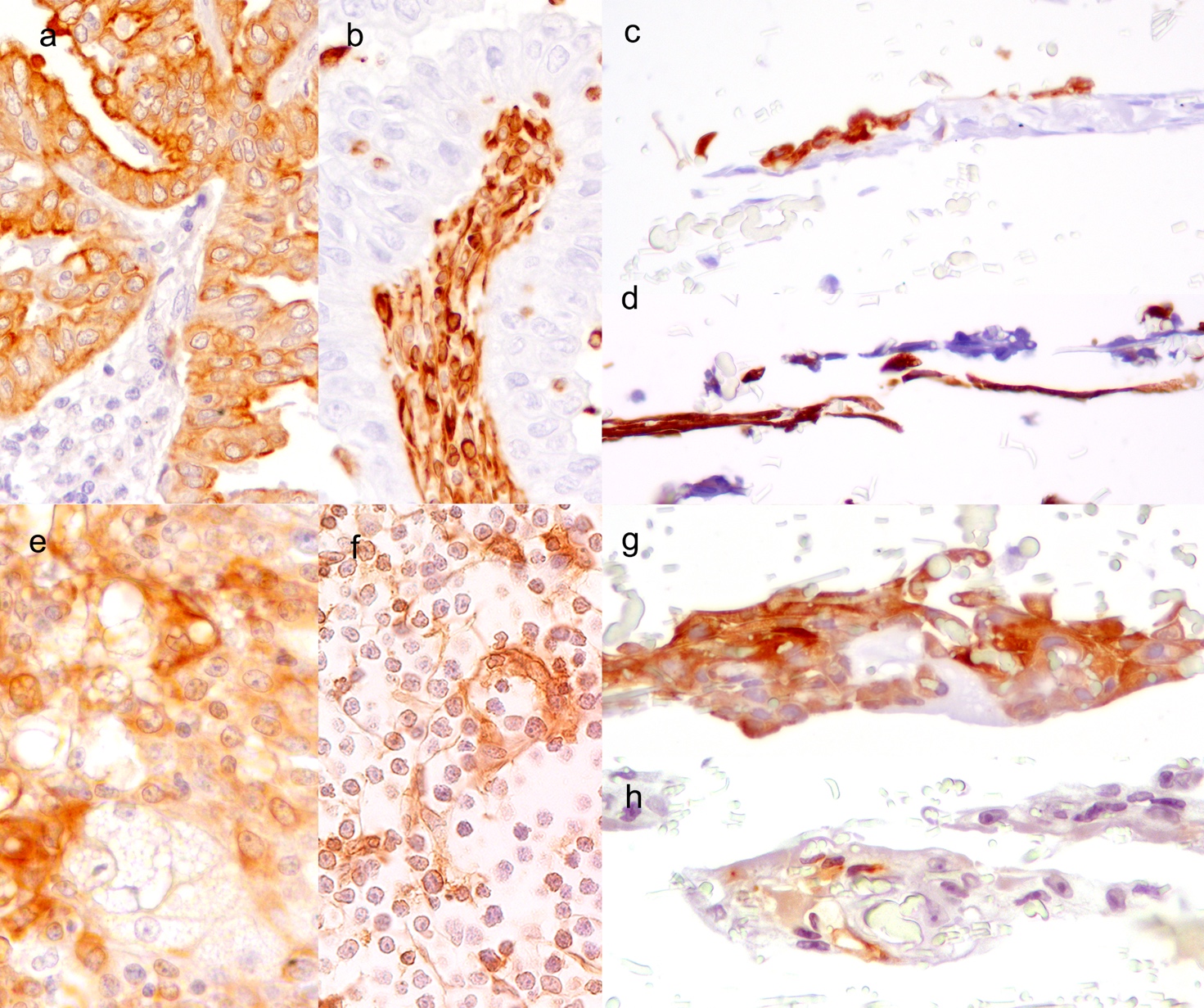
**

**Supplementary Figure 1.** Stromal component and immune cells in primary tumors and 3D cultures, diaminobenzidine, 400x. (a) Pulmonary carcinoma, pancytokeratin: papillae of neoplastic cells positive stained for pancytokeratin are supported by a stromal axis composed of negative labelled mesenchymal cells. (b) Pulmonary carcinoma, vimentin: papillae of neoplastic cells negative stained for vimentin are supported by a stromal axis composed of mesenchymal cells with intense diffuse cytoplasmic immunoexpression. (c) 3D culture of pulmonary carcinoma, pancytokeratin: positive labelled epithelial cells are supported by an axis of negative stained spindle cells, suggestive of stromal support. (d) 3D culture of pulmonary carcinoma, vimentin: supporting positive stained mesenchymal cells are underlined to negative stained neoplastic cells. (e) Epitheliomatous sebaceous carcinoma, pancytokeratin: solid lobules of neoplastic cells positive stained for pancytokeratin with cytoplasmic expression. (f) Epitheliomatous sebaceous carcinoma, CD18: scattered leucocytes infiltrating the neoplasm are stained positive with membranous to cytoplasmic expression. (g) 3D culture of epitheliomatous sebaceous carcinoma, pancytokeratin: solid lobules of neoplastic cells positive stained for pancytokeratin with cytoplasmic expression. (h) 3D culture of epitheliomatous sebaceous carcinoma, CD18: scattered macrophages are present, recapitulating the primary tumor immune-microenviroment.
